# Vascular smooth muscle cell loss underpins the accelerated atherosclerosis in Hutchinson-Gilford progeria syndrome

**DOI:** 10.1080/19491034.2019.1589359

**Published:** 2019-03-22

**Authors:** Magda R. Hamczyk, Vicente Andrés

**Affiliations:** a Laboratory of Molecular and Genetic Cardiovascular Pathophysiology, Vascular Pathophysiology Area, Centro Nacional de Investigaciones Cardiovasculares (CNIC), Madrid, Spain; b CIBER de Enfermedades Cardiovasculares (CIBERCV), Spain; c Departamento de Bioquímica y Biología Molecular, Universidad de Oviedo, Oviedo, Spain

**Keywords:** Atherosclerosis, Hutchinson-Gilford progeria syndrome, lamin A, progerin, vascular smooth muscle cells

## Abstract

Lamin A, a product of the *LMNA* gene, is an essential nuclear envelope component in most differentiated cells. Mutations in *LMNA* have been linked to premature aging disorders, including Hutchinson-Gilford progeria syndrome (HGPS). HGPS is caused by progerin, an aberrant form of lamin A that leads to premature death, typically from the complications of atherosclerotic disease. A key characteristic of HGPS is a severe loss of vascular smooth muscle cells (VSMCs) in the arteries. Various mouse models of HGPS have been created, but few of them feature VSMC depletion and none develops atherosclerosis, the death-causing symptom of the disease in humans. We recently generated a mouse model that recapitulates most features of HGPS, including VSMC loss and accelerated atherosclerosis. Furthermore, by generating cell-type–specific HGPS mouse models, we have demonstrated a central role of VSMC loss in progerin-induced atherosclerosis and premature death.

## Characteristics of Hutchinson-Gilford progeria syndrome (HGPS)

HGPS is a premature aging disease that was first described by Jonathan Hutchinson in 1886 and Hastings Gilford in 1897 [1,2]. The prevalence of HGPS is about 1 in 20 million and it has no ethnic or gender bias [3]. Affected children appear normal at birth but progressively develop signs of premature aging, including alopecia, mottled and wrinkled skin, loss of body fat, and joint and bone abnormalities (osteoporosis and osteolysis of the mandible, phalanges, and clavicles) [3,4]. A key feature of HGPS is early-onset cardiovascular disease (CVD), leading to death typically from myocardial infarction or stroke at a mean age of 14.6 years [5]. While HGPS recapitulates most features of physiological aging, patients show no signs of neurodegenerative disease or cancer [3,5].

Atherosclerosis underlies death in most HGPS patients, and autopsy examination has revealed a wide variety of plaque phenotypes, ranging from early- to late-stage lesions [3,6]. Atherosclerotic lesions in HGPS usually exhibit calcification and signs of plaque erosion or rupture, similar to what happens in normal aging [6]. The severity of atherosclerosis in progeria typically correlates with the loss of medial vascular smooth muscle cells (VSMCs), an event that coincides with increased extracellular matrix (ECM) deposition and altered elastin structure [6–8]. HGPS arteries and veins also show pronounced thickening of the adventitia, and this, together with the VSMC loss and excessive ECM accumulation, can result in vessel stiffening and reduced vascular compliance [6]. Furthermore, HGPS patients show age-related defects in cardiac conduction, manifested as prolonged QT interval, ST segment depression/elevation, and negative or biphasic T waves on electrocardiography (ECG) [4,9]. Interestingly, although they exhibit accelerated atherosclerosis, most HGPS patients lack typical cardiovascular risk factors, such as hypercholesterolemia and increased serum C-reactive protein [10]. Thus, progeria provides a model of cardiovascular aging isolated from classical cardiovascular risk factors and can enhance our understanding of vascular stiffening, atherosclerosis, and downstream cardiovascular events such as myocardial infarction and stroke [11,12].

## Genetic and molecular mechanisms causing HGPS

Although HGPS was first described at the end of the 19^th^ century [1,2], the cause of the disease remained elusive until 2003, when Eriksson et al. [13] and De Sandre-Giovannoli et al. [14] reported that HGPS patients carry a heterozygous *de novo* point mutation in the *LMNA* gene. *LMNA* encodes A-type lamins, which are nuclear envelope proteins involved in maintaining nuclear structure and function [15]. The two major A-type lamin variants are lamin A and lamin C and arise from alternative splicing of *LMNA* mRNA [16]. Unlike lamin C, lamin A is formed through several posttranslational modifications of its precursor form, prelamin A. First, the CaaX motif located at the C-terminus is farnesylated [17,18]. Next, the last three residues are removed, and the newly exposed C-terminal cysteine is carboxymethylated [18]. Finally, the zinc metallopeptidase STE24 (ZMPSTE24) cleaves the last 15 amino acids, together with the farnesyl and carboxymethyl groups, resulting in mature lamin A [19–21].

HGPS is typically caused by a cytosine-to-thymine substitution in exon 11 of *LMNA* (c.1824C>T; p.G608G); this mutation does not affect the encoded amino acid but instead activates a cryptic splice site, leading to aberrant splicing between exons 11 and 12 [13,14]. The resulting mRNA is 150 nucleotides shorter than native *LMNA* mRNA, and results in the expression of progerin, a truncated form of lamin A that lacks 50 amino acids, including the cleavage site for ZMPSTE24 near the C-terminus. Progerin therefore remains permanently farnesylated and carboxymethylated. Because of the continued presence of the farnesyl group, progerin, unlike lamin A, stays permanently anchored in the inner nuclear membrane and leads to abnormalities in nuclear shape and function [22,23]. Since lamin A is involved in numerous cellular processes, progerin expression has effects on a wide range of functions, including gene expression, signal transduction, heterochromatin organization, cell cycle, and DNA repair [15, 24].

## An atherosclerosis-susceptible mouse model with ubiquitous progerin expression

Several progerin-expressing mouse models have been generated for the study of HGPS, including G608G BAC mice [25], *Lmna^HG/HG^* mice [26,27], and *Lmna^G609G/G609G^* mice [28,29]. HGPS has also been studied in prelamin A-expressing *Zmpste24*^-/-^ mice [20,30], whose phenotype closely resembles type B mandibuloacral dysplasia [31]. The phenotypes of these mice are diverse, probably reflecting the different gene modification strategies used, which result in varying levels of progerin (or prelamin A), as well as the presence or absence of other *Lmna* gene products. Among these models, the *Lmna^G609G/G609G^* knock-in mice generated by Osorio et al. [28] carry a point mutation in the *Lmna* gene (c.1827C>T; p.G609G); as occurs in HGPS patients, this mutation leads to the production of progerin via aberrant splicing. Homozygous *Lmna^G609G/G609G^* mice show growth retardation, loss of subcutaneous fat, attrition of hair follicles, bone alterations, and a shortened lifespan (a mean of 4 months, versus more than 2 years in wild-type controls) [28]. Notably, these mice present moderate VSMC depletion in the medial layer of the aortic arch, bradycardia, and alterations in cardiac electrical conduction. However, like other HGPS models, *Lmna^G609G/G609G^* mice do not develop atherosclerosis, even when fed a high-fat diet [32]. This is in line with numerous studies showing that mice are relatively resistant to atherosclerosis due to profound differences versus humans in their lipid profile; mice have high levels of anti-atherosclerotic high-density lipoproteins (HDLs) and low levels of the pro-atherosclerotic low-density lipoproteins (LDLs) and very low-density lipoproteins (VLDLs) [33]. Atherosclerosis development in mice therefore usually requires genetic manipulation to alter lipid metabolism, often in combination with the use of western-type diets enriched in cholesterol and saturated fat.

One of the most commonly-used mouse models of atherosclerosis is the *Apoe* knockout [34–36]. The *Apoe* gene encodes apolipoprotein E (Apo-E), a key component of plasma lipoproteins, including chylomicrons, VLDLs, and HDLs [37,38]. Apo-E is an important ligand for lipoprotein uptake by hepatocytes. Most circulating Apo-E is produced in the liver, but considerable amounts are also synthesized in the brain, kidneys, adipocytes, and macrophages [39–42]. Defective lipoprotein clearance in *Apoe*-deficient mice results in elevated plasma levels of atherogenic lipoproteins. These mice therefore develop atherosclerosis on a standard chow diet, and the disease is more severe when the animals are fed western-type high-fat diets [43,44]. Nevertheless, these mice rarely develop the complications observed in human atherosclerosis, such as plaque rupture and ensuing myocardial infarction or stroke.

To generate a model for the study of atherosclerosis in the context of progeria, we crossed *Lmna^G609G^* mice with *Apoe*-deficient mice [32]. As anticipated, *Apoe^-/-^Lmna^G609G/G609G^* mice with ubiquitous progerin expression showed signs of premature aging and had lower body weight and survival than *Apoe^-/-^* littermates expressing wild-type lamin A/C. Importantly, *Apoe^-/-^Lmna^G609G/G609G^* mice exhibited accelerated atherosclerosis development in the aorta. The increase in lesion formation was more prominent in older mice, fat-fed mice, and, remarkably, in the thoracic aorta, the aortic region usually least prone to atherosclerosis. Fat-fed *Apoe^-/-^Lmna^G609G/G609G^* mice presented exacerbated lipid accumulation in areas of the aortic media that were typically devoid of VSMCs and rich in ECM, including regions of the media without neighboring atherosclerotic plaques. Aortas of *Apoe^-/-^Lmna^G609G/G609G^* mice also had a thickened and dense adventitial layer, a hallmark of vascular disease in HGPS patients [6]. Notably, atherosclerotic lesions in *Apoe^-/-^Lmna^G609G/G609G^* mice presented vulnerable plaque features, such as large necrotic cores, presence of erythrocytes and iron deposits, and reduced VSMC content in the fibrous cap. Additional cardiovascular alterations in *Apoe^-/-^Lmna^G609G/G609G^* animals included bradycardia, prolonged QRS, QT, and QTc intervals, and an elevated incidence of arrhythmias. The *Apoe^-/-^Lmna^G609G/G609G^* mice thus featured many of the cardiovascular alterations observed in HGPS and, similarly to patients, showed no serum cholesterol elevation versus control *Apoe^-/-^* mice.

## An atherosclerosis-susceptible mouse model with VSMC-specific progerin expression

Atherosclerosis is a disease of the arteries that leads to their hardening and narrowing with age [12,45]. The disease is usually initiated by LDL accumulation in the arterial wall, causing endothelial cell (EC) dysfunction and leukocyte recruitment to the inflamed site [46]. Recruited monocytes differentiate into macrophages, which upon ingesting LDLs convert into foam cells that secrete pro-inflammatory molecules and attract more immune cells to the growing atherosclerotic lesion. Failure to process the ingested lipoproteins can result in foam cell death and accumulation of debris and cholesterol crystals in the intima, leading to the formation of a necrotic core. Signaling from the growing intima induces phenotypic changes in VSMCs and causes their proliferation and migration from the media to the intima [47]. VSMCs typically migrate towards the subendothelial space to form a fibrous cap, which protects the plaque from rupture and prevents exposure of pro-thrombotic necrotic core constituents to the blood.

Given the central role of ECs, leukocytes, and VSMCs in atherosclerosis development during normal aging, we reasoned that these cell types might contribute to progerin-driven premature atherosclerosis. Analysis of human and mouse HGPS arterial tissue identified VSMCs and, to a lesser extent, macrophages as possible contributors to accelerated atherosclerosis [6,32]. We therefore developed mouse models in which progerin expression was restricted to either VSMCs (*Apoe^-/-^Lmna^LCS/LCS^SM22αCre*) or macrophages (*Apoe^-/-^Lmna^LCS/LCS^LysMCre*) [32]. To generate cell-type–specific HGPS mice, we used a knock-in *Lmna^LCS/LCS^* (Lamin C-Stop) model, which expresses only lamin C [28]. When crossed with a Cre-expressing line, *Lmna^LCS/LCS^* mice express progerin and lamin C (and a residual level of lamin A) in the tissue or cell type of interest. The phenotype of *Lmna^LCS/LCS^* mice is mild, including slightly higher body weight and longer survival than wild-type mice with normal lamin A/C expression [48]. We also observed a non-statistically significant trend toward protection against atherosclerosis in atherosclerosis-prone *Apoe^-/-^Lmna^LCS/LCS^* mice compared with *Apoe^-/-^* controls [32].

VSMC- and macrophage-specific progeroid mouse models appeared normal at birth and showed no overt aging phenotype. However, the *Apoe^-/-^Lmna^LCS/LCS^SM22αCre* mice stopped gaining weight at around 5 months of age and died suddenly between 6.5 and 16.5 months of age, whereas the *Apoe^-/-^Lmna^LCS/LCS^LysMCre* mice had normal body weight throughout life and a normal lifespan. Compared with control *Apoe^-/-^Lmna^LCS/LCS^* mice, fat-fed *Apoe^-/-^Lmna^LCS/LCS^SM22αCre* mice (but not *Apoe^-/-^Lmna^LCS/LCS^LysMCre* mice) had a higher atherosclerosis burden and aortic structural alterations (VSMC loss, adventitial thickening, and lipid accumulation in the media). Moreover, atherosclerotic plaques in *Apoe^-/-^Lmna^LCS/LCS^SM22αCre* mice had vulnerable features, thus fully recapitulating the vascular disease observed in the ubiquitous progeria model. Remarkably, all these vascular pathologies were independent of the elevated serum cholesterol, which was indistinguishable from the level in *Apoe^-/-^Lmna^LCS/LCS^* mice. Unlike *Apoe^-/-^Lmna^G609G/G609G^* mice, *Apoe^-/-^Lmna^LCS/LCS^SM22αCre* mice presented no ECG alterations at 4 months of age, consistent with the VSMC-specificity of the model.

## Cause of death in progeria mouse models

Although the ubiquitous and VSMC-specific progeroid models had the same vascular phenotype, *Apoe^-/-^Lmna^LCS/LCS^SM22αCre* mice showed none of the premature aging features and cardiac alterations observed in *Apoe^-/-^Lmna^G609G/G609G^* mice (Table 1) [32]. Moreover, while both models had a shortened lifespan, survival of *Apoe^-/-^Lmna^LCS/LCS^SM22αCre* mice was almost double that of *Apoe^-/-^Lmna^G609G/G609G^* mice. Insight into the cause of death in these animals was gained by further comparison between atherosclerosis-prone progeroid mice (*Apoe^-/-^Lmna^G609G/G609G^* and *Apoe^-/-^Lmna^LCS/LCS^SM22αCre*) and atheroresistant progeroid mice with an intact *Apoe* gene (*Lmna^G609G/G609G^* and *Lmna^LCS/LCS^SM22αCre*). Survival was similar in atherosclerosis-prone *Apoe^-/-^Lmna^G609G/G609G^* mice and atherosclerosis-free *Lmna^G609G/G609G^* mice, suggesting that the cause of death is the same in both models and is independent of atherosclerosis. In contrast, whereas lifespan in atherosclerosis-free *Lmna^LCS/LCS^SM22αCre* mice was the same as in control mice, atherosclerosis-prone *Apoe^-/-^Lmna^LCS/LCS^SM22αCre* mice died prematurely. These findings suggest that atherosclerosis underlies death in *Apoe^-/-^Lmna^LCS/LCS^SM22αCre* mice but not in *Apoe^-/-^Lmna^G609G/G609G^* mice. Histopathological analysis of aorta and heart samples from both models confirmed that the stage of CVD in mice close to maximum lifespan is compatible with death from atherosclerosis-related causes in *Apoe^-/-^Lmna^LCS/LCS^SM22αCre* mice but not in *Apoe^-/-^Lmna^G609G/G609G^* mice. Thus, death in *Apoe^-/-^Lmna^G609G/G609G^* mice is likely due to other disease symptoms arising before atherosclerosis becomes a life-threatening symptom, such as arrhythmias, cachexia, and starvation [9,49]. In ubiquitous progeria mice, progerin is expressed in most differentiated cells (following the pattern of lamin A expression); consequently, CVD development and premature aging and death in these animals likely involve contributions from many cell types, including cardiomyocytes, fibroblasts, ECs, and VSMCs. Supporting this view, Osmanagic-Myers et al. recently reported that transgenic mice with EC-specific progerin expression present myocardial fibrosis associated with left ventricular hypertrophy and diastolic dysfunction [50], cardiac pathologies that may also be involved in premature death in progeria.

**Table 1.** Comparison of phenotypic features between atherosclerosis-prone mouse models with ubiquitous and vascular smooth muscle cell-specific progerin expression.

Feature	*Apoe^-/-^Lmna^G609G/G609G^*	*Apoe^-/-^Lmna^LCS/LCS^SM22αCre*	
Appear aged	yes	no	Aging phenotype and lifespan
Shortened lifespan	yes (median survival ≈18 weeks)	yes (median survival ≈34 weeks)	
Thickened adventitia	yes	yes	**Vascular phenotype** (at 16 weeks of age on a high-fat diet and, to a lesser extent, on normal chow diet)
VSMC loss in the media	yes	yes	
Lipid retention in the media	yes	yes	
Increased atherosclerosis	yes	yes	
Vulnerable plaque features	yes	yes	
Bradycardia	yes	no	**Cardiac phenotype** (at 16 weeks of age on normal chow diet)
Arrhythmia	yes	no	
Prolonged QRS, QT, QTc	yes	no	

Even though the principal cause of death in *Apoe^-/-^Lmna^G609G/G609G^* and *Apoe^-/-^Lmna^LCS/LCS^SM22αCre* mice is different, both models showed evidence of plaque disruption and myocardial infarction [32], complications rarely seen in mice but typical of late stages of atherosclerosis in HGPS and non-HGPS individuals. These findings, together with the VSMC loss and adventitial thickening observed in these mice, demonstrate that *Apoe^-/-^Lmna^G609G/G609G^* and *Apoe^-/-^Lmna^LCS/LCS^SM22αCre* models faithfully model the vascular disease found in HGPS patients.

## How does progerin expression in VSMCs accelerate atherosclerosis?

Studies with *Apoe^-/-^Lmna^LCS/LCS^SM22αCre* mice have demonstrated that progerin expression in VSMCs is sufficient to cause progressive loss of these cells and accelerate atherosclerotic disease in the absence of cholesterol elevation relative to *Apoe^-/-^Lmna^LCS/LCS^* control mice [32]. Progerin-triggered VSMC death was accompanied by excessive lipid accumulation in the aortic wall, suggesting that increased retention of atherogenic LDLs may account, at least in part, for the early onset of atherosclerosis in *Apoe^-/-^Lmna^G609G/G609G^* and *Apoe^-/-^Lmna^LCS/LCS^SM22αCre* mice. Experiments with 16-week-old mice fed normal chow and injected with fluorescently-labelled LDLs showed enhanced LDL retention in the aortic wall in both the ubiquitous and the VSMC-specific progeroid models. At this age, aortas of *Apoe^-/-^Lmna^G609G/G609G^* and *Apoe^-/-^Lmna^LCS/LCS^SM22αCre* mice show moderate loss of VSMCs and their replacement by ECM, suggesting that increased ECM content or altered ECM composition promotes LDL retention. However, it remains to be determined whether premature atherosclerosis in HGPS also involves contributions from other factors, such as increased endothelial permeability resulting from endothelial dysfunction.

## Concluding remarks

*Apoe^-/-^* mice with ubiquitous and VSMC-specific progerin expression offer a unique opportunity to study atherosclerosis in the setting of progeria. *Apoe^-/-^Lmna^G609G/G609G^* mice, which show both a premature aging phenotype and accelerated CVD, permit the study of progeria features in a broader context. Conversely, *Apoe^-/-^Lmna^LCS/LCS^SM22αCre* mice allow investigation of progerin-induced vascular disease, including atherosclerosis, in isolation from other premature aging symptoms. Moreover, these models can aid in the understanding of physiological aging [11,12], since small amounts of progerin are also produced in the cells of normally-aging non-HGPS individuals [6,51,52]. Given that the expression of progerin in VSMCs plays a crucial role in accelerated atherosclerosis and premature death in *Apoe^-/-^* mice, further efforts are warranted to reveal the mechanisms underlying VSMC loss in HGPS. These studies may also shed light on progeria-unrelated diseases characterized by VSMC loss, such as aneurysm formation.
